# New solvent options for *in vivo* assays in the *Galleria mellonella* larvae model

**DOI:** 10.1080/21505594.2019.1659663

**Published:** 2019-08-26

**Authors:** Beatriz Suay-García, Pedro A. Alemán-López, José Ignacio Bueso-Bordils, Antonio Falcó, Gerardo Antón-Fos, María Teresa Pérez-Gracia

**Affiliations:** a Departamento de Farmacia, Instituto de Ciencias Biomédicas, Facultad de Ciencias de la Salud. Universidad Cardenal Herrera-CEU, Valencia, España; b ESI International Chair@CEU-UCH. Departamento de Matemáticas, Física y Ciencias Tecnológicas. Universidad Cardenal Herrera-CEU, Valencia, España

**Keywords:** Solvent, *in vivo* assay, toxicity, *Galleria mellonella*, animal model

## Abstract

Experimentation in mammals is a long and expensive process in which ethical aspects must be considered, which has led the scientific community to develop alternative models such as that of *Galleria mellonella*. This model is a cost and time effective option to act as a filter in the drug discovery process. The main limitation of this model is the lack of variety in the solvents used to administer compounds, which limits the compounds that can be studied using this model. Five aqueous (DMSO, MeOH, acetic acid, HCl and NaOH) and four non-aqueous (olive oil, isopropyl myristate, benzyl benzoate and ethyl oleate) solvents was assessed to be used as vehicles for toxicity and antimicrobial activity *in vivo* assays. All the tested solvents were innocuous at the tested concentrations except for NaOH, which can be used at a maximum concentration of 0.5 M. The toxicity of two additional compounds, 5-aminosalicylic acid and DDT, was also assessed. The results obtained allow for the testing of a broader range of compounds using wax moth larvae. This model appears as an alternative to mammal models, by acting as a filter in the drug development process and reducing costs and time invested in new drugs.

## Introduction

When a new drug proves to be effective *in vitro*, it is important to study *in vivo* toxicity and activity, for which animal models are normally employed [1,2]. In these *in vivo* studies, the new drug is tested both, for the desired activity in order to identify any factors that may limit said activity, and on its own to determine whether its toxicity prevents its potential application in humans [2].

Generally, *in vivo* assays are carried out in rodent models, seeing as they have a similar metabolism to that of humans. However, mammal experimentation is a long and expensive process in which certain ethical aspects must be considered. In fact, the 3Rs (Replacement, Reduction and Refinement) have become a working standard for high quality scientific production in the academic and industrial sector, focusing on the development of alternative models that reduce the use of animals [3]. With these disadvantages in mind, non-mammal models have been developed which, by being cheaper and not having to take any ethical considerations into account, can be applied as a first filter, thus reducing mammal use and the global cost associated to drug development. In fact, it has been proven that plant, insect, nematode and cell culture-based models provide interesting data at a reduced cost and without the ethical requirements related to mammals [4–7].

Among these alternative models, the *Galleria mellonella* model is gaining popularity because, unlike other non-mammal models, it allows for both, toxicity and antimicrobial activity testing [8–14]. This is due to the fact that larvae survive at 37 °C [15]. *Galleria mellonella* is an insect from de *Lepidoptera* order. Last instar larvae are used for testing, which can be obtained from the egg in 5 weeks. The larvae have a weight of 180–350 mg, reaching 250–300 mm in length, large enough for an easy injection for intraperitoneal administration [16]. Currently, *Galleria mellonella* larvae have been used as an infection model against bacteria, fungi and virus [17].

For these reasons, *G. mellonella* larvae are an ideal model to act as a first step previous to testing in mammals, seeing as those compounds that prove to be inactive or toxic in the larval model would not continue to mammal tests, thus reducing expenses and time, as well as avoiding the ethical limitations.

When studying the toxicity of a compound, it can be administered pure as a liquid in different amounts or dissolved in a solvent that is not toxic at different concentrations. Moreover, when determining the antimicrobial activity of a compound, the chosen vehicle must be innocuous and have no antimicrobial activity at the concentration used [18,19].

Having such a versatile model for *in vivo* testing, it is interesting to know the toxicity of different solvents in this model, seeing as there is not enough information regarding the effect of aqueous vehicles at different pH values or non-aqueous solvents in these larvae. This limits compound administration considerably, seeing as any product that is not soluble at a neutral pH or is only soluble in non-polar solvents cannot be tested using this model.

The aim of this article is to identify the dose of different solvents that can be administered in these larvae without them being toxic or showing antimicrobial activity in order to use them as vehicles in *in vivo* assays using this model. Additionally, the experimental protocol used by different research groups when working with *G. mellonella* larvae has been briefly reviewed.

## Materials and methods

### Solvent and compound selection

A total of 9 solvents that are commonly used for *in* vitro [19] and *in vivo* [20] antibacterial activity studies were selected. Of these, 5 were aqueous solutions of basic, acidic and neutral solvents (aqueous solutions of DMSO, MeOH, acetic acid, HCl and NaOH) and 4 non-polar solvents (olive oil, isopropyl myristate, benzyl benzoate and ethyl oleate).

Furthermore, two additional compounds were chosen for further studies in order to demonstrate the usefulness of this study: 5-aminosalicylic acid and dichlorophenyltrichloroethane (DDT).

All the solvents were obtained from Acros Organics (Geel, Belgium). 5-Aminosalicylic acid and DDT were obtained from Sigma-Aldrich (Madrid, Spain). All of the solvents and compounds were used without further purification

### Solution preparation

Sterile distilled water (SDW) was used to prepare the aqueous solutions of the 5 polar solvents. 5-aminosalicylic acid was dissolved using SDW to prepare a stock solution for each of the doses administered to the larvae. Regarding DDT, the stock solutions were prepared using olive oil, while the suspensions were prepared with SDW. Table 1 shows the concentrations of the stock solutions prepared for each dose.

**Table 1.** Concentrations of the stock solutions for the administered doses.

Dose (mg/kg)	Concentration (mg/mL)
50	1.5
75	2.25
125	3.75
150	4.5
300	9

### Toxicity assays using G. mellonella larvae

In order to carry out the tests, last instar *G. mellonella* larvae were acquired from TruLarv (BioSystems Technology, Exeter, UK). The average weight was of 300 mg ± 20 mg. These larvae are research grade approved and antimicrobial and hormone free.

Firstly, larvae were kept in the dark at 15 °C before the assays in order to slow down their growth [21]. Assays were carried out using 5 larvae per dose studied and repeating each test three times [22]. One control group was used in which the larvae were punctured with the needle in order to dismiss possible deaths related to injection trauma.

Compounds were administered via injection through the last pro-leg using a 10 µL 26g Hamilton™ Microliter syringes and a total injection volume of 10 µL. Once injected, larvae were kept at 30°C during the observation period.

For result interpretation, the larvae were observed every 24 h for 5 days, sufficient time for the compound to show its effect in the larva without it evolving into its next phase. Larvae are considered to be dead when there was no reaction after placing it face up or touching it and if the body blackens. Results were analyzed using the probit method to provide the compound’s median lethal dose (LD_50_) in mg kg^−1^, in order to compare results with those obtained using other *in vivo* models.

Once the tests had concluded, any surviving larvae were eliminated by keeping them at −20°C during 2 h before disposal.

## Results

Out of the 9 solvents selected for their toxicity assessment in the *G. mellonella* larvae model, it was observed that all were innocuous at the tested concentrations except for NaOH (Table 2). The administration of an aqueous solution of NaOH caused the deaths of half of the larvae when 10 µL of NaOH 1 M were injected, which translates to a LD_50_ of 1.3 mg/kg. As a result, all of the tested solvents are suitable to be used for the administration of compounds in these larvae except for NaOH, which can be used as long as the concentration of NaOH does not exceed 0.5 M.

**Table 2.** Toxicity of the different solvents tested in *G. mellonella* larvae.

Solvent	Concentrations^a^	Observations	Reccomendation
DMSO	10, 20 and 30%	Innocuous	Suitable for use up to 30%
MeOH	10, 20 and 30%	Innocuous	Suitable for use up to 30%
Acetic acid	10, 20 and 30%	Innocuous	Suitable for use up to 30%
HCl	0.5, 1 and 2.5 M	Innocuous	Innocuous, consider its effects in antibacterial activity studies
NaOH	0.5, 1 and 2.5 M	LD_50_ = 1.3mg/kg	Do not exceed a 0.5 M concentration
Olive oil	Pure	Innocuous	Suitable to be used pure
Isopropylmyristate	Pure	Innocuous	Suitable to be used pure
Benzyl benzoate	Pure	Innocuous	Suitable to be used pure
Ethyl oleate	Pure	Innocuous	Suitable to be used pure

^a^Aqueous solutions.

Regarding the chosen doses, DMSO, MeOH and acetic acid were not studied at higher concentrations because they could show antimicrobial activity, which could alter the results obtained. However, HCl and NaOH were tested at higher concentrations because these would be the media used to dissolve any compound during toxicity assays.

Having broadened the solvent options for the parenteral administration of compounds to *G. mellonella* larvae and in order to prove its usefulness, two additional chemical compounds were selected to study their toxicity using this model (Table 3). 5-Aminosalicylic acid was administered as a solution in aqueous NaOH 0.5 M, showing a calculated LD_50_ of 254 mg/kg. On the other hand, DDT was administered as both a solution in olive oil and a suspension in water. In this case, both experiments resulted in a calculated LD_50_ of 121.8 mg/kg.

**Table 3.** Toxicity of the different chemical compounds tested in *G. mellonella* larvae.

Compound	Assayed doses(mg/kg)	Solvent	Toxicity (LD_50_ in mg/kg)
5-aminosalicylic acid	50, 125 and 300	NaOH 0.5M	254
DDT	50, 75 and 150	Olive oil	121.8
DDT	50, 75 and 150	H_2_O^a^	121.8

aDDT is insoluble in H_2_O, in this case the compound was administered as a suspension.

## Discussion

The use of *G. mellonella* larvae as an *in vivo* model is very recent, reason for which all its applications have not been fully developed. It is interesting to note the studies by Ignasiak et al. and Allegra et al., seeing as both works to determine the correlation between this new model and the traditional rodent model [23,24]. Ignasiak et al. studied the correlation between rodent and *G. mellonella* models for toxicity and antibacterial activity assays. Their results show that antibacterial activity can be studied *in vivo* using the larvae, seeing as the dose recommended for humans proved to be effective in systemically infected larvae [23]. Moreover, the study concludes that there is also a good correlation in the toxicity of the compounds studied in larvae and rodents. Along these lines, Allegra et al. studied the ability of the model to differentiate toxic and non-toxic compounds [24]. Results showed that, out of the 19 compounds studied, the wax moth larvae classified 11 correctly according to the GHS classification (Globally Harmonized System of Classification and Labeling of Chemicals) measured in rats. These studies confirm the usefulness of larvae as a first screening step before testing in mammals.

Interestingly, due to the novelty of this model, there is scarce information regarding the optimal experimental conditions to be used in this model. Tables 4 and 5 collect the experimental conditions used by different authors for antimicrobial activity and toxicity testing in this model respectively. As in can be observed in both tables, the solvents used so far were limited to PBS, aqueous solutions of DMSO below 20% and water. Evidently, this limited the compounds that could be tested using this model, as any compound insoluble in these three vehicles could not be tested; especially bearing in mind that there are no studies on the effect of injecting suspended compounds in these larvae. Therefore, having confirmed the innocuousness of 9 more solvents greatly increases the possibilities to use this model during drug development.

**Table 4.** Conditions for antimicrobial activity testing in *G. mellonella.*

Microorganism	Tested compound	Solvent	Injected volume (µl)	Larvae no.	Repetitions	Injected CFUs**	Observation time (h)	Temp (°C)	References
*C. albicans*	Fluconazole Tetracycline	PBS	10	20	3	4 × 10^6^	96	37	Gu et al. 2017 [8]
	Triazole-amino acid hybrids	PBS + DMSO 12.5%	20	10	NS*	5 × 10^5^	24	30	Aneja et al. 2016 [9]
	1,5 and 2,5-disubstituted tetrazoles	PBS	10	10	3	NS*	48	NS*	Staniszewska et al. 2018 [10]
	Tetrazole derivatives	PBS	10	10	NS*	NS*	96	35	Lukowska et al. 2016 [11]
	Silver (I) complexes of 9-anthracenecarboxylic acids	10% DMSO	20	10	NS*	1 × 10^8^	72	NS*	McCann et al. 2012 [27]
*S. aureus*	Daptomycin, penicillin and vancomycin	PBS	10	10	NS*	1 × 10^6^	48	NS*	Desbois et al. 2011 [28]
	Anti-MRSA Protonophore	PBS	10	12	NS*	2 × 10^6^	120	37	Tharmalingam et al. 2017 [29]
*Paracoccidioides spp.*	Amphotericin B Itraconazole	PBS	10	16	3	5 × 10^6^	168	37	Lacorte et al. 2016 [12]
*A. baumannii*	Theaflavin and epicatechin	PBS	10	10	3	1 × 10^5^	96	37	Betts et al. 2017 [13]
*C. gattii*	3ʹ-hydro × ychalcone	NS	10	10	3	1 × 10^6^	168	37	Palanco et al. 2017 [14]
*S. aureus* *C. albicans*	Carbene silver (I) acetate derivative	H_2_O + 5% DMSO	20	10	3	1 × 10^6^	24	30	Browne et al. 2014 [30]
*E. coli* *M. smegmatis* *P. aeruginosa* *S. aureus*	Ampicillin Ciproflo × acin Tetracycline Rifampicin	H_2_O or DMSO	10	5	3	5 × 10^5^	120	37	Ignasiak et al. 2017 [23]
*C. neoformans*	Amphotericin B Fluconazole Flucytosine	H_2_O	10	12–16	NS*	1.5 × 10^4^	NS*	37	Mylonakis et al. 2005 [31]

*NS: not specified **CFUs: colony-forming units

**Table 5.** Conditions for toxicity testing in *G. mellonella.*

Tested compound	Dose or concentration	Solvent	Injected volume (µl)	Larva nº	Repetitions	Observation time (h)	Temp. (°C)	References
Arsenic, DDT, Ethylene dichloride, Nicotine, Parathion	NS*	H_2_O or Olive oil	1	4751**	NS*	NS*	NS*	Beard 1949 [25]
Anticolinesterases	NS*	Olive oil	NS*	NS*	NS*	NS*	NS*	Beard 1951 [26]
4-arylcoumarins	NS*	20% DMSO	1	20	3	NS*	NS*	Zhang et al. 2014 [32]
1-alkyl-3-methylimidazolium chloride ionic liquids	NS*	H_2_O	20	10	1	24	30	Megaw et al. 2015 [33]
Potassium nitrite, Sodium nitrite, Sodium benzoate, Potassium sorbate, Sodium acetate, Sodium nitrate, Potassium nitrate, NaCl	5.0 × 10^−6^, 1.0 × 10^−6^, 1.5 × 10^−6^, 2.0 × 10^−6^, 2.5 × 10^−6^, 3.0 × 10^−6^ M	NS*	20	10	NS*	48	30	Maguire et al. 2016 [34]
Caffeine	1.52 × 10^−5^ M	PBS	20	10	NS*	24	30	Maguire et al. 2017 [35]
Amsacrine, Chloroquine, Ciprofloxacin, DMSO, Doxorubicin, Etoposide, Glucose, Novobiocin, NaCl, Streptomycin, Tetracycline	5, 50, 125, 300, 1000, 2000 mg kg^−1^	H_2_O or 10% DMSO	10	5	3	120	NS*	Ignasiak et al. 2017 [23]
Disulfoton, cadmium chloride, triphenyltin hydroxide, Phenol, Digoxin, Atropine sulfate Colchicine, Lindane, Acetaminophen, citric acid, trichloroacetic acid, sodium hypochlorite, sodium dichromate dihydrate, procainamide HCl, 2-propanol, dibutyl phthalate, Dimethylformamide, Glycerol, Acetonitrile	6 dilutions***	H_2_O, DMSO or EtOH	10	10	NS*	72	20–25	Allegra et al. 2018 [24]

a NS: Not specified; **Total number of larvae used; ***Specific concentrations are not detailed.

It should be noted that Beard [25,26] described the use of olive oil as a vehicle for the administration of compounds in *G. mellonella*, however, those studies were focused on insecticide activity rather than *in vivo* toxicity assessment as such. In fact, the only other condition defined in these studies is the administration volume of 1 µL, which differs greatly from the conditions defined by other authors.

While analyzing the solvents used by different authors, a lack of standardization in the experimental protocol was also detected. Tables 4 and 5 show how there is great variability in the number of larvae used for each assay, the observation time after injection, the temperature at which larvae are kept during the study, the injection volume and the number of times each assay is repeated.

Regarding the number of larvae used for each experimental condition, studies range between 5 and 20 larvae per test; being 10 the most frequently used number of larvae. However, it must be noted that, in order to be able to compare results obtained using this model with results obtained from rodent models with greater precision, the OECD (Organization for Economic Cooperation and Development) guidelines recommend using 5 animals per assay [22]. The same can be said about observation time post-infection which, as Tables 1 and 2 show, varies between 24 and 168h. Even though most studies have an observation period of 96 h our less, OECD guidelines recommend an observation period of 120 h.

The temperature at which the larvae are kept after the tested compound is injected is crucial, as it affects their development; the higher the temperature, the more its life cycle accelerates [21]. For antibacterial activity assays, temperature oscillates between 30 and 37 °C, although most studies keep the larvae at 37 °C in order to optimize bacterial growth. As for toxicity assays, several authors keep the larvae at 30 °C, and many others do not specify the conditions at which the larvae are kept post-injection. Likewise, not all authors specify the number of times assays are repeated for a given dose or concentration. Similarly, there is no consensus regarding injection volume. Bibliography suggests an injection volume ranging from 10 to 20 µL, bearing in mind that this could be doubled in antibacterial activity assays where each larva must be injected twice (bacterial inoculation and compound administration).

Having analyzed the experimental procedures followed in different studies that use *G. mellonella* larvae, we designed our own protocol as described in the materials and methods section. In this case, we chose to use 5 larvae for each dose, as established by the OECD guidelines [22]. Similarly, we also adopted the observation period of 120 h suggested by these same guidelines. As for the temperature at which the larvae were kept after the inoculation, we decided to keep them at 30 °C, seeing as it is a temperature at which results can be observed within the observation period without the larvae evolving into the next stage of its life cycle. Regarding injection volume, we used 10 µL because we found that higher volumes affected larval mobility and would not allow for a second injection in the case of antimicrobial activity assays. Finally, we repeated every assay three times in order to assure the reproducibility of the results obtained.

One additional fact that should be taken into account with the solvents assessed in this paper is that, even if they are innocuous for the larvae, they can alter the results of certain activity tests. This is the case with HCl, which cannot be used at a concentration higher than 0.05 mol/L in antimicrobial activity studies, seeing as the observed antibacterial effect could be due to the solvent and not to the studied compound [18]. Regardless, it can still be used at a concentration of 2.5 M for toxicity assays (Table 2). Similarly, DMSO, MeOH and acetic acid cannot be used at concentrations higher than 1% of the final volume for DMSO and MeOH and 2.5 µL/mL for acetic acid for antimicrobial activity assays due to the possible antibacterial effect of the solvent. However, in all 3 cases, the concentrations described in Table 2 as innocuous can be used for toxicity studies.

Having broadened the selection of vehicles available for compound injection in *G. mellonella* larvae, we decided to test two additional compounds. Firstly, we selected 5-aminosalicylic acid because it had been described by other authors as “not applicable” in this model due to its bad solubility [24]. However, this compound is easily soluble in NaOH 0.5 M (Table 3) thus, it could be tested with the new data available. Having been able to determine the toxicity of this compound proves the usefulness of the results obtained in this paper.

Moreover, there could still be cases in which the targeted compound is insoluble in all the available solvents. In this case, we considered the option of injecting a suspension of the compound in the larvae as it had been described by other authors [9]. However, this study [9] does not compare results with the administration of the same compound in a solution, therefore, there is no data regarding the effect this may have on the final results. The oral administration of suspensions in these larvae has been described but the effects of a parenteral injection remain unknown [36]. For this reason and, having learned the lack of toxicity of olive oil in the wax moth larvae (Table 2), we decided to assess if there were any notable differences between the administration of the same compound in suspension or solution using DDT. As it can be observed by the results obtained, there are no notable differences between the results of both experiments (Table 3). However, further studies should be carried out to confirm this observation.

This study has broadened the options for the parenteral administration of compounds to *G. mellonella* larvae by assessing the toxicity/innocuousness of 5 aqueous and 4 non-aqueous vehicles in this model. These solvents allow the dilution of most compounds in order to carry out *in vivo* assays using this model. This, along with the preliminary data obtained regarding the injection of compounds in suspension, allows for the testing of a broader range of compounds using wax moth larvae. Furthermore, the different experimental conditions used in studies with these larvae have been reviewed. With these new data, the *G. mellonella* larvae *in vivo* model appears as a very interesting alternative to mammal models, which could act as a filter in the drug development process and, thus, reduce costs and time invested in new active molecules.
